# Supplementary figures and images for: Characterization of prophages in bacterial genomes from the honey bee (Apis mellifera) gut microbiome
Source: PeerJ. 2023 Jun 9;11:e15383. doi: 10.7717/peerj.15383 (PMC10259446; doi:10.7717/peerj.15383)

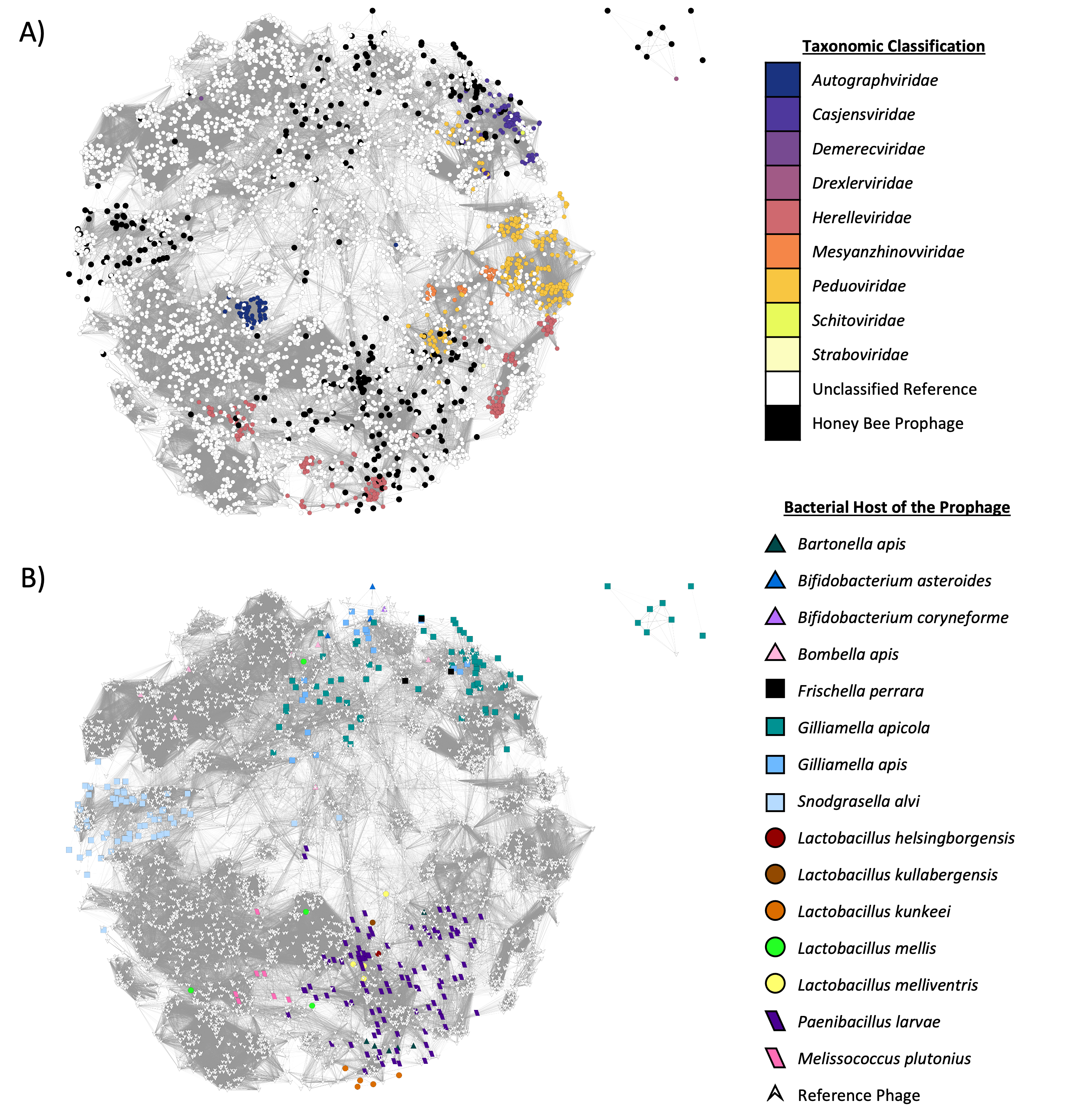

Supplement: Figure S1 — A vConTACT2 network created with both honey bee isolated prophages and reference phage from the INPHARED database (January 2023). Visually, only reference phage that are third neighbors or less to a honey bee prophage are represented. Nodes indicate individual phages and the opacity of edges represent the strength of shared gene interactions. (A) Honey bee isolated prophages were unable to be taxonomically classified by vConTACT2, but often clustered near other taxonomically classified reference phage. Larger black circles are prophages identified from honey bee symbionts, while smaller colored circles depict the taxonomic family of reference phages. (B) Honey bee isolated prophages (host indicated by shape and color) tended to cluster near other phage from the same bacterial host, but were not spatially separated from reference phages. [file peerj-11-15383-s001.png]
